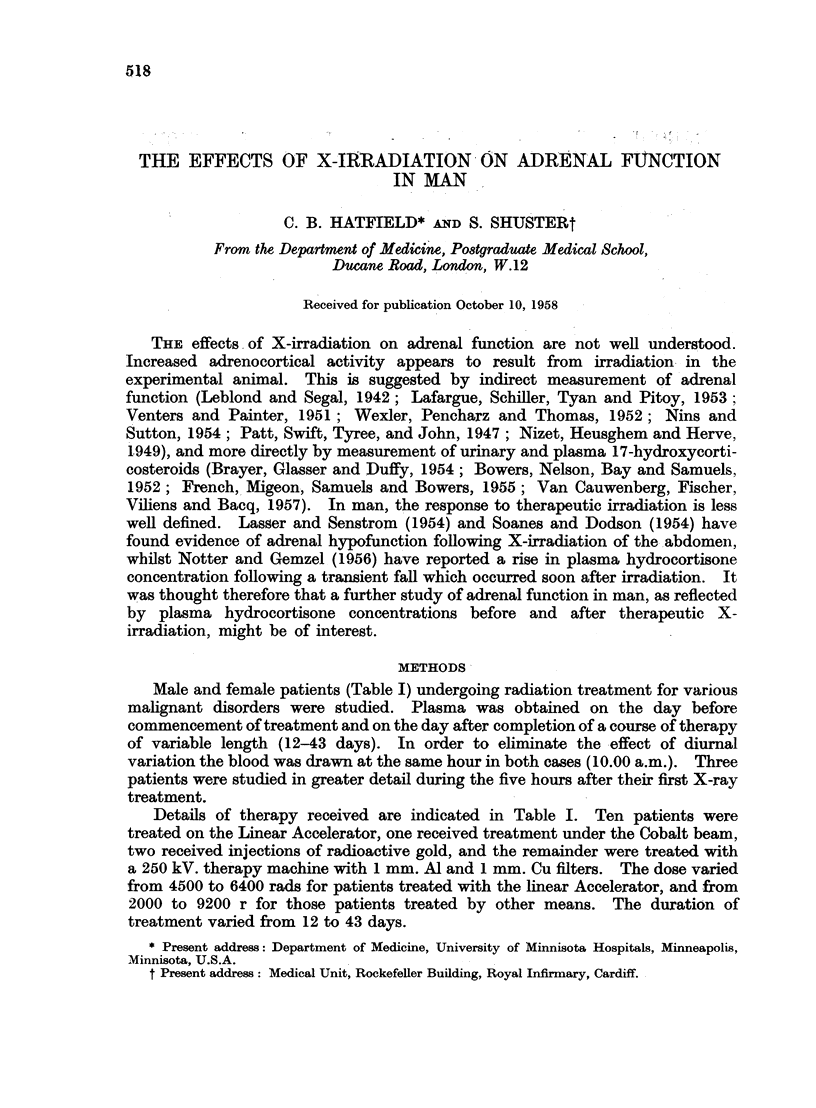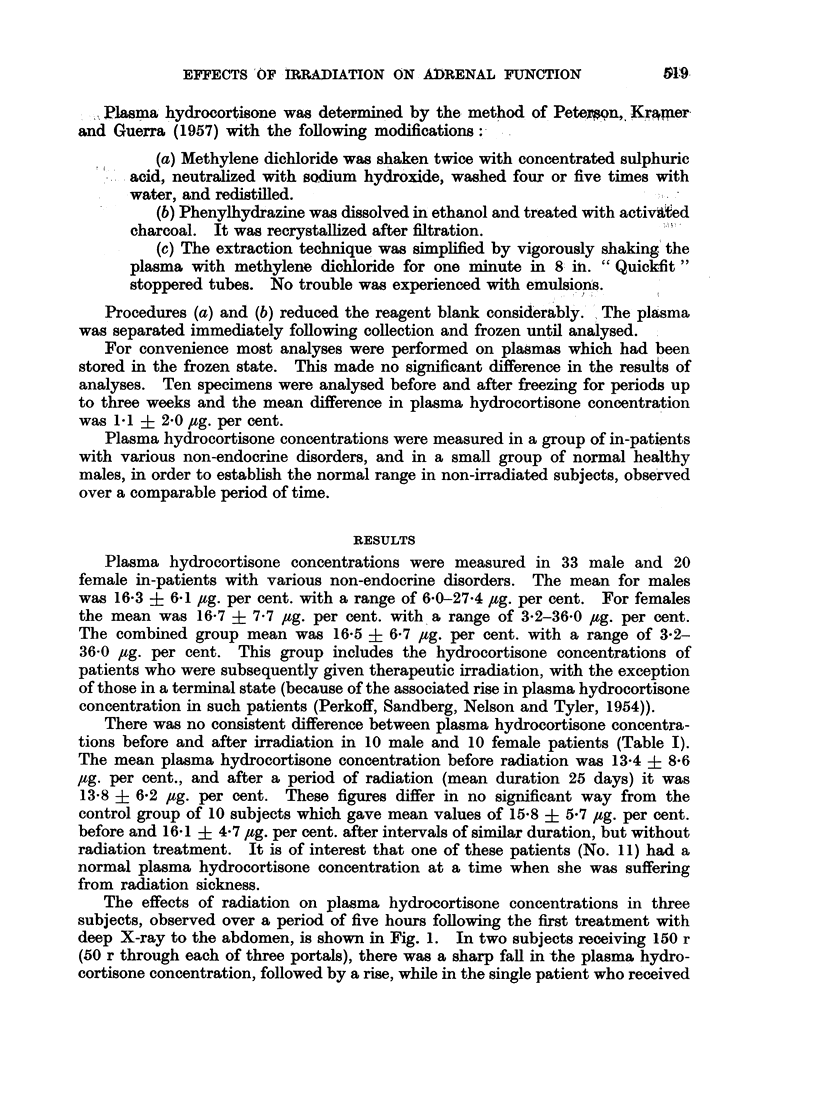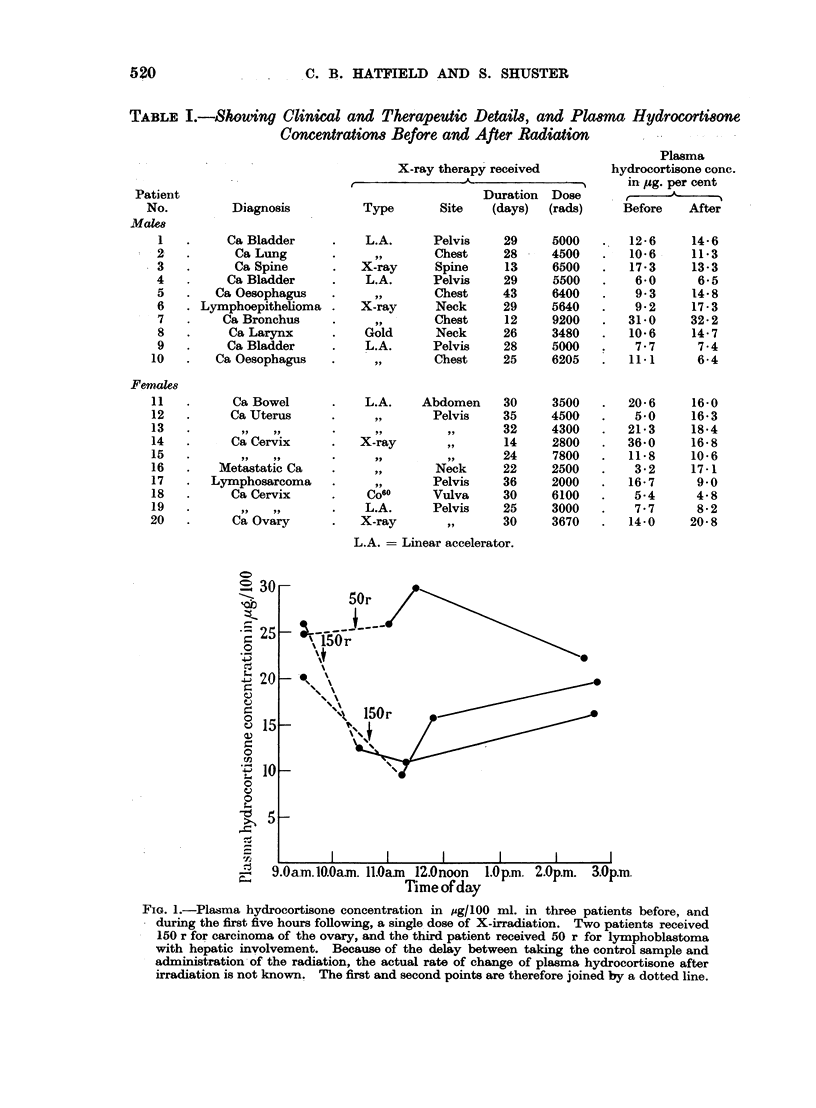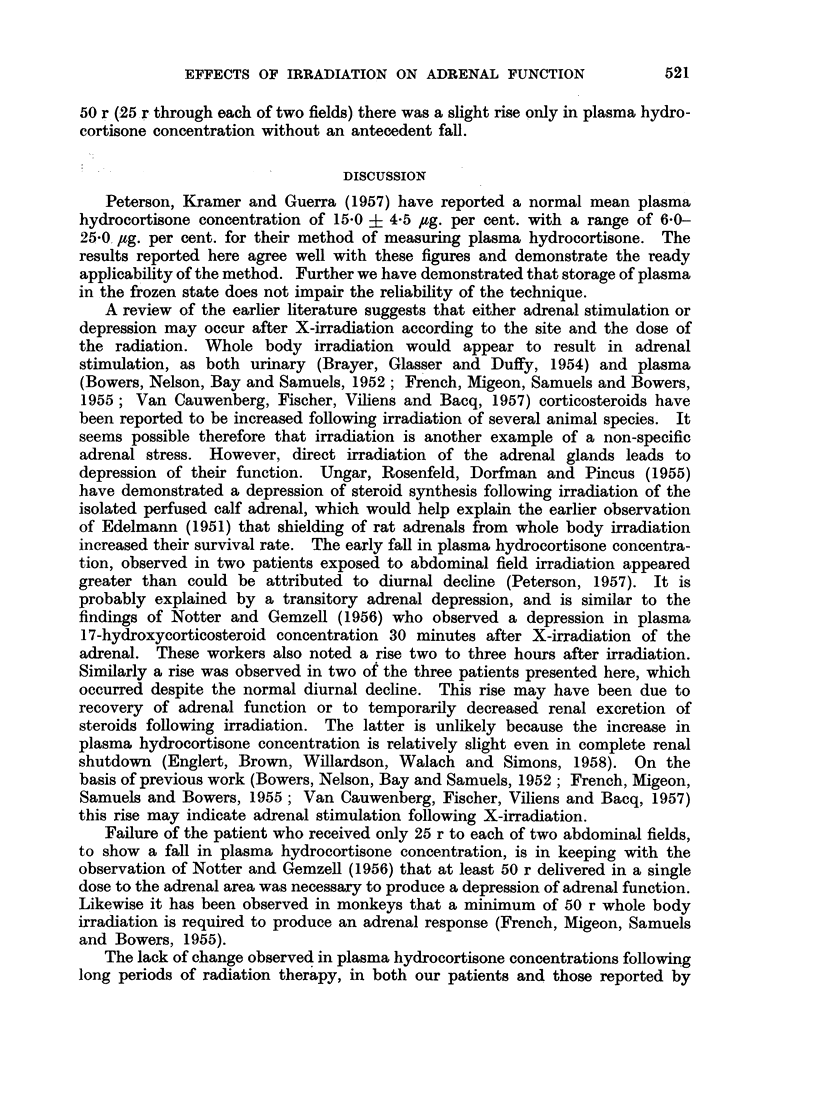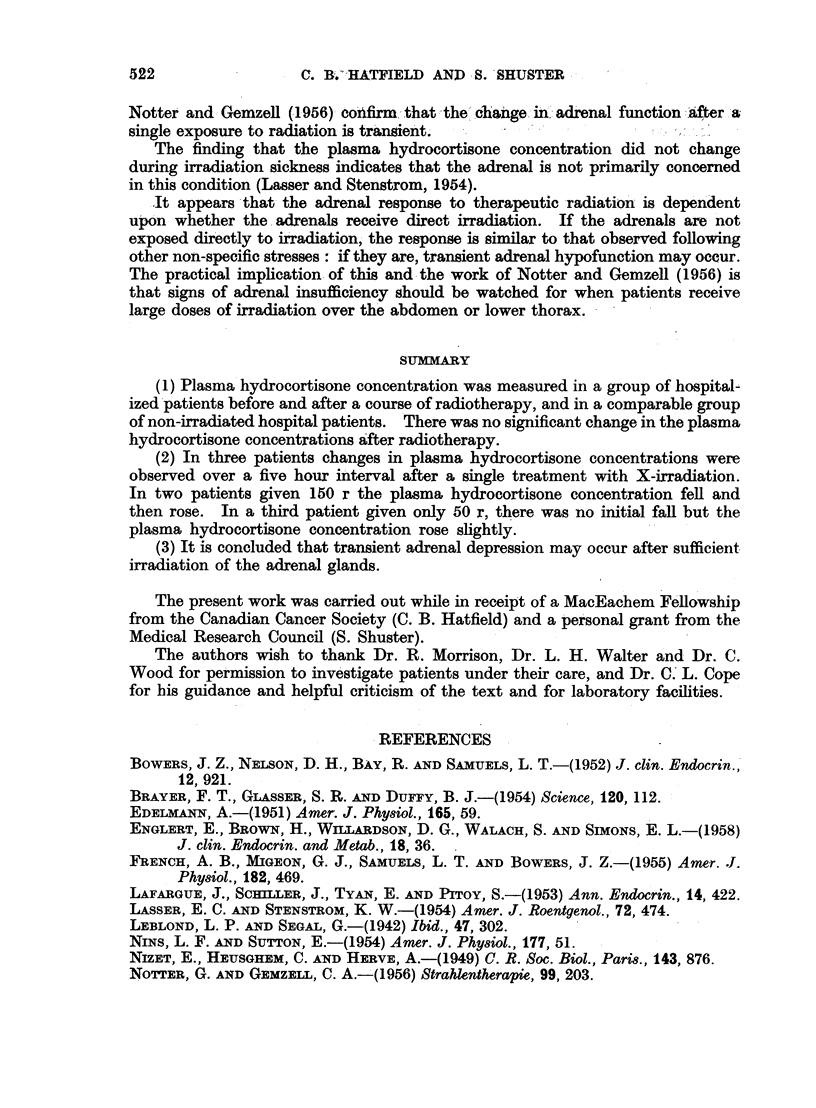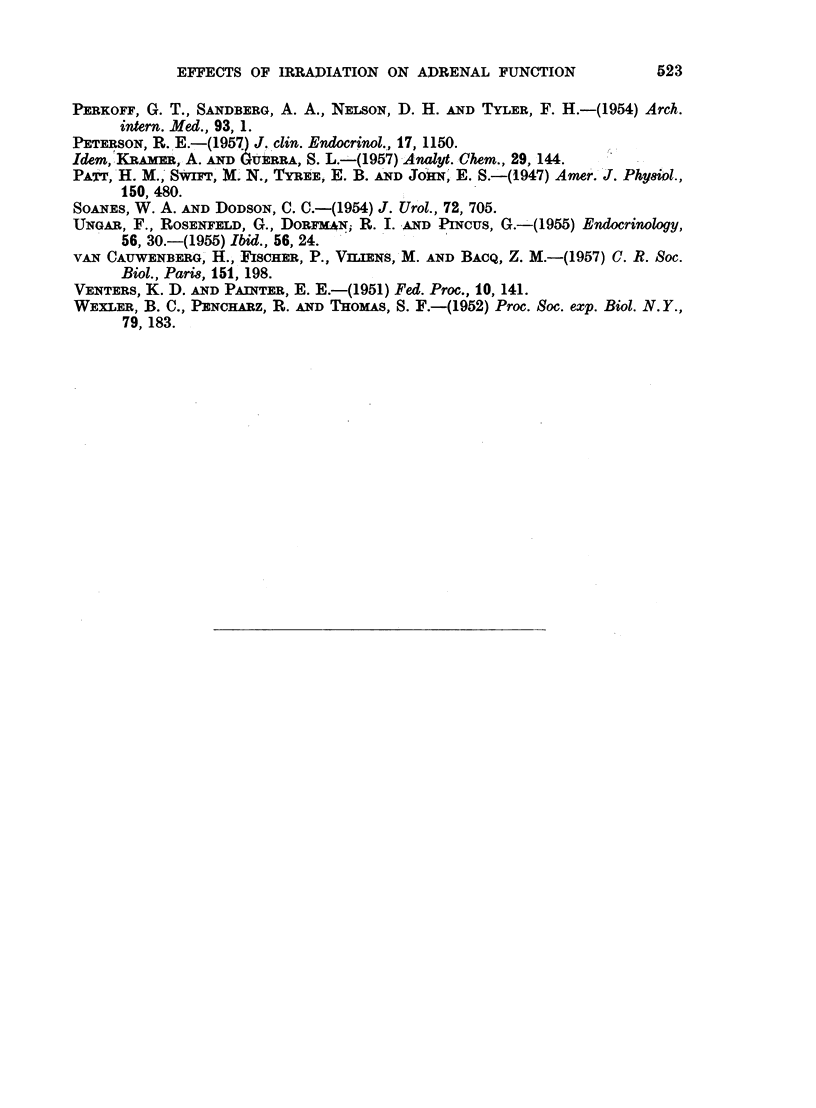# The Effects Of X-Irradiation on Adrenal Function in Man

**DOI:** 10.1038/bjc.1958.60

**Published:** 1958-12

**Authors:** C. B. Hatfield, S. Shuster


### THE EFFECTS OF X-IRRADIATION ON ADRENAL FUNCTION IN MAN

C. B. HATFIELD* AND S. SHUSTER†

*From the Department of Medicine, Postgraduate Medical School, Ducane Road, London, W.12*

Received for publication October 10, 1958

THE effects of X-irradiation on adrenal function are not well understood. Increased adrenocortical activity appears to result from irradiation in the experimental animal. This is suggested by indirect measurement of adrenal function (Leblond and Segal, 1942 ; Lafargue, Schiller, Tyan and Pitoy, 1953 ; Venters and Painter, 1951 ; Wexler, Pencharz and Thomas, 1952 ; Nins and Sutton, 1954 ; Patt, Swift, Tyree, and John, 1947 ; Nizet, Heusghem and Herve, 1949), and more directly by measurement of urinary and plasma 17-hydroxycorticosteroids (Brayer, Glasser and Duffy, 1954 ; Bowers, Nelson, Bay and Samuels, 1952 ; French, Migeon, Samuels and Bowers, 1955 ; Van Cauwenberg, Fischer, Viliens and Bacq, 1957). In man, the response to therapeutic irradiation is less well defined. Lasser and Senstrom (1954) and Soanes and Dodson (1954) have found evidence of adrenal hypofunction following X-irradiation of the abdomen, whilst Notter and Gemzel (1956) have reported a rise in plasma hydrocortisone concentration following a transient fall which occurred soon after irradiation. It was thought therefore that a further study of adrenal function in man, as reflected by plasma hydrocortisone concentrations before and after therapeutic X-irradiation, might be of interest.

### METHODS

Male and female patients (Table I) undergoing radiation treatment for various malignant disorders were studied. Plasma was obtained on the day before commencement of treatment and on the day after completion of a course of therapy of variable length (12–43 days). In order to eliminate the effect of diurnal variation the blood was drawn at the same hour in both cases (10.00 a.m.). Three patients were studied in greater detail during the five hours after their first X-ray treatment.

Details of therapy received are indicated in Table I. Ten patients were treated on the Linear Accelerator, one received treatment under the Cobalt beam, two received injections of radioactive gold, and the remainder were treated with a 250 kV. therapy machine with 1 mm. Al and 1 mm. Cu filters. The dose varied from 4500 to 6400 rads for patients treated with the linear Accelerator, and from 2000 to 9200 r for those patients treated by other means. The duration of treatment varied from 12 to 43 days.

* Present address: Department of Medicine, University of Minnisota Hospitals, Minneapolis, Minnisota, U.S.A.

† Present address : Medical Unit, Rockefeller Building, Royal Infirmary, Cardiff.

Plasma hydrocortisone was determined by the method of Peterson, Kramer and Guerra (1957) with the following modifications :

(*a*) Methylene dichloride was shaken twice with concentrated sulphuric acid, neutralized with sodium hydroxide, washed four or five times with water, and redistilled.

(*b*) Phenylhydrazine was dissolved in ethanol and treated with activated charcoal. It was recrystallized after filtration.

(*c*) The extraction technique was simplified by vigorously shaking the plasma with methylene dichloride for one minute in 8 in. " Quickfit " stoppered tubes. No trouble was experienced with emulsions.

Procedures (*a*) and (*b*) reduced the reagent blank considerably. The plasma was separated immediately following collection and frozen until analysed.

For convenience most analyses were performed on plasmas which had been stored in the frozen state. This made no significant difference in the results of analyses. Ten specimens were analysed before and after freezing for periods up to three weeks and the mean difference in plasma hydrocortisone concentration was 1·1 ± 2·0 μg. per cent.

Plasma hydrocortisone concentrations were measured in a group of in-patients with various non-endocrine disorders, and in a small group of normal healthy males, in order to establish the normal range in non-irradiated subjects, observed over a comparable period of time.

### RESULTS

Plasma hydrocortisone concentrations were measured in 33 male and 20 female in-patients with various non-endocrine disorders. The mean for males was 16·3 ± 6·1 μg. per cent. with a range of 6·0–27·4 μg. per cent. For females the mean was 16·7 ± 7·7 μg. per cent. with a range of 3·2–36·0 μg. per cent. The combined group mean was 16·5 ± 6·7 μg. per cent. with a range of 3·2–36·0 μg. per cent. This group includes the hydrocortisone concentrations of patients who were subsequently given therapeutic irradiation, with the exception of those in a terminal state (because of the associated rise in plasma hydrocortisone concentration in such patients (Perkoff, Sandberg, Nelson and Tyler, 1954)).

There was no consistent difference between plasma hydrocortisone concentrations before and after irradiation in 10 male and 10 female patients (Table I). The mean plasma hydrocortisone concentration before radiation was 13·4 ± 8·6 μg. per cent., and after a period of radiation (mean duration 25 days) it was 13·8 ± 6·2 μg. per cent. These figures differ in no significant way from the control group of 10 subjects which gave mean values of 15·8 ± 5·7 μg. per cent. before and 16·1 ± 4·7 μg. per cent. after intervals of similar duration, but without radiation treatment. It is of interest that one of these patients (No. 11) had a normal plasma hydrocortisone concentration at a time when she was suffering from radiation sickness.

The effects of radiation on plasma hydrocortisone concentrations in three subjects, observed over a period of five hours following the first treatment with deep X-ray to the abdomen, is shown in Fig. 1. In two subjects receiving 150 r (50 r through each of three portals), there was a sharp fall in the plasma hydrocortisone concentration, followed by a rise, while in the single patient who received

TABLE I.—*Showing Clinical and Therapeutic Details, and Plasma Hydrocortisone Concentrations Before and After Radiation*

| Patient No. | Diagnosis | X-ray therapy received: Type | Site | Duration (days) | Dose (rads) | Plasma hydrocortisone conc. in μg. per cent: Before | After |
|---|---|---|---|---|---|---|---|
| *Males* | | | | | | | |
| 1 | Ca Bladder | L.A. | Pelvis | 29 | 5000 | 12·6 | 14·6 |
| 2 | Ca Lung | ,, | Chest | 28 | 4500 | 10·6 | 11·3 |
| 3 | Ca Spine | X-ray | Spine | 13 | 6500 | 17·3 | 13·3 |
| 4 | Ca Bladder | L.A. | Pelvis | 29 | 5500 | 6·0 | 6·5 |
| 5 | Ca Oesophagus | ,, | Chest | 43 | 6400 | 9·3 | 14·8 |
| 6 | Lymphoepithelioma | X-ray | Neck | 29 | 5640 | 9·2 | 17·3 |
| 7 | Ca Bronchus | ,, | Chest | 12 | 9200 | 31·0 | 32·2 |
| 8 | Ca Larynx | Gold | Neck | 26 | 3480 | 10·6 | 14·7 |
| 9 | Ca Bladder | L.A. | Pelvis | 28 | 5000 | 7·7 | 7·4 |
| 10 | Ca Oesophagus | ,, | Chest | 25 | 6205 | 11·1 | 6·4 |
| *Females* | | | | | | | |
| 11 | Ca Bowel | L.A. | Abdomen | 30 | 3500 | 20·6 | 16·0 |
| 12 | Ca Uterus | ,, | Pelvis | 35 | 4500 | 5·0 | 16·3 |
| 13 | ,, ,, | ,, | ,, | 32 | 4300 | 21·3 | 18·4 |
| 14 | Ca Cervix | X-ray | ,, | 14 | 2800 | 36·0 | 16·8 |
| 15 | ,, ,, | ,, | ,, | 24 | 7800 | 11·8 | 10·6 |
| 16 | Metastatic Ca | ,, | Neck | 22 | 2500 | 3·2 | 17·1 |
| 17 | Lymphosarcoma | ,, | Pelvis | 36 | 2000 | 16·7 | 9·0 |
| 18 | Ca Cervix | $Co^{60}$ | Vulva | 30 | 6100 | 5·4 | 4·8 |
| 19 | ,, ,, | L.A. | Pelvis | 25 | 3000 | 7·7 | 8·2 |
| 20 | Ca Ovary | X-ray | ,, | 30 | 3670 | 14·0 | 20·8 |

L.A. = Linear accelerator.

FIG. 1.—Plasma hydrocortisone concentration in μg/100 ml. in three patients before, and during the first five hours following, a single dose of X-irradiation. Two patients received 150 r for carcinoma of the ovary, and the third patient received 50 r for lymphoblastoma with hepatic involvement. Because of the delay between taking the control sample and administration of the radiation, the actual rate of change of plasma hydrocortisone after irradiation is not known. The first and second points are therefore joined by a dotted line.

50 r (25 r through each of two fields) there was a slight rise only in plasma hydrocortisone concentration without an antecedent fall.

### DISCUSSION

Peterson, Kramer and Guerra (1957) have reported a normal mean plasma hydrocortisone concentration of 15·0 ± 4·5 μg. per cent. with a range of 6·0–25·0 μg. per cent. for their method of measuring plasma hydrocortisone. The results reported here agree well with these figures and demonstrate the ready applicability of the method. Further we have demonstrated that storage of plasma in the frozen state does not impair the reliability of the technique.

A review of the earlier literature suggests that either adrenal stimulation or depression may occur after X-irradiation according to the site and the dose of the radiation. Whole body irradiation would appear to result in adrenal stimulation, as both urinary (Brayer, Glasser and Duffy, 1954) and plasma (Bowers, Nelson, Bay and Samuels, 1952 ; French, Migeon, Samuels and Bowers, 1955 ; Van Cauwenberg, Fischer, Viliens and Bacq, 1957) corticosteroids have been reported to be increased following irradiation of several animal species. It seems possible therefore that irradiation is another example of a non-specific adrenal stress. However, direct irradiation of the adrenal glands leads to depression of their function. Ungar, Rosenfeld, Dorfman and Pincus (1955) have demonstrated a depression of steroid synthesis following irradiation of the isolated perfused calf adrenal, which would help explain the earlier observation of Edelmann (1951) that shielding of rat adrenals from whole body irradiation increased their survival rate. The early fall in plasma hydrocortisone concentration, observed in two patients exposed to abdominal field irradiation appeared greater than could be attributed to diurnal decline (Peterson, 1957). It is probably explained by a transitory adrenal depression, and is similar to the findings of Notter and Gemzell (1956) who observed a depression in plasma 17-hydroxycorticosteroid concentration 30 minutes after X-irradiation of the adrenal. These workers also noted a rise two to three hours after irradiation. Similarly a rise was observed in two of the three patients presented here, which occurred despite the normal diurnal decline. This rise may have been due to recovery of adrenal function or to temporarily decreased renal excretion of steroids following irradiation. The latter is unlikely because the increase in plasma hydrocortisone concentration is relatively slight even in complete renal shutdown (Englert, Brown, Willardson, Walach and Simons, 1958). On the basis of previous work (Bowers, Nelson, Bay and Samuels, 1952 ; French, Migeon, Samuels and Bowers, 1955 ; Van Cauwenberg, Fischer, Viliens and Bacq, 1957) this rise may indicate adrenal stimulation following X-irradiation.

Failure of the patient who received only 25 r to each of two abdominal fields, to show a fall in plasma hydrocortisone concentration, is in keeping with the observation of Notter and Gemzell (1956) that at least 50 r delivered in a single dose to the adrenal area was necessary to produce a depression of adrenal function. Likewise it has been observed in monkeys that a minimum of 50 r whole body irradiation is required to produce an adrenal response (French, Migeon, Samuels and Bowers, 1955).

The lack of change observed in plasma hydrocortisone concentrations following long periods of radiation therapy, in both our patients and those reported by

Notter and Gemzell (1956) confirm that the change in adrenal function after a single exposure to radiation is transient.

The finding that the plasma hydrocortisone concentration did not change during irradiation sickness indicates that the adrenal is not primarily concerned in this condition (Lasser and Stenstrom, 1954).

It appears that the adrenal response to therapeutic radiation is dependent upon whether the adrenals receive direct irradiation. If the adrenals are not exposed directly to irradiation, the response is similar to that observed following other non-specific stresses : if they are, transient adrenal hypofunction may occur. The practical implication of this and the work of Notter and Gemzell (1956) is that signs of adrenal insufficiency should be watched for when patients receive large doses of irradiation over the abdomen or lower thorax.

SUMMARY

(1) Plasma hydrocortisone concentration was measured in a group of hospitalized patients before and after a course of radiotherapy, and in a comparable group of non-irradiated hospital patients. There was no significant change in the plasma hydrocortisone concentrations after radiotherapy.

(2) In three patients changes in plasma hydrocortisone concentrations were observed over a five hour interval after a single treatment with X-irradiation. In two patients given 150 r the plasma hydrocortisone concentration fell and then rose. In a third patient given only 50 r, there was no initial fall but the plasma hydrocortisone concentration rose slightly.

(3) It is concluded that transient adrenal depression may occur after sufficient irradiation of the adrenal glands.

The present work was carried out while in receipt of a MacEachem Fellowship from the Canadian Cancer Society (C. B. Hatfield) and a personal grant from the Medical Research Council (S. Shuster).

The authors wish to thank Dr. R. Morrison, Dr. L. H. Walter and Dr. C. Wood for permission to investigate patients under their care, and Dr. C. L. Cope for his guidance and helpful criticism of the text and for laboratory facilities.

### REFERENCES

BOWERS, J. Z., NELSON, D. H., BAY, R. AND SAMUELS, L. T.—(1952) *J. clin. Endocrin.*, **12**, 921.

BRAYER, F. T., GLASSER, S. R. AND DUFFY, B. J.—(1954) *Science*, **120**, 112.

EDELMANN, A.—(1951) *Amer. J. Physiol.*, **165**, 59.

ENGLERT, E., BROWN, H., WILLARDSON, D. G., WALACH, S. AND SIMONS, E. L.—(1958) *J. clin. Endocrin. and Metab.*, **18**, 36.

FRENCH, A. B., MIGEON, G. J., SAMUELS, L. T. AND BOWERS, J. Z.—(1955) *Amer. J. Physiol.*, **182**, 469.

LAFARGUE, J., SCHILLER, J., TYAN, E. AND PITOY, S.—(1953) *Ann. Endocrin.*, **14**, 422.

LASSER, E. C. AND STENSTROM, K. W.—(1954) *Amer. J. Roentgenol.*, **72**, 474.

LEBLOND, L. P. AND SEGAL, G.—(1942) *Ibid.*, **47**, 302.

NINS, L. F. AND SUTTON, E.—(1954) *Amer. J. Physiol.*, **177**, 51.

NIZET, E., HEUSGHEM, C. AND HERVE, A.—(1949) *C. R. Soc. Biol., Paris.*, **143**, 876.

NOTTER, G. AND GEMZELL, C. A.—(1956) *Strahlentherapie*, **99**, 203.

Perkoff, G. T., Sandberg, A. A., Nelson, D. H. and Tyler, F. H.—(1954) *Arch. intern. Med.*, **93**, 1.
Peterson, R. E.—(1957) *J. clin. Endocrinol.*, **17**, 1150.
*Idem*, Kramer, A. and Guerra, S. L.—(1957) *Analyt. Chem.*, **29**, 144.
Patt, H. M., Swift, M. N., Tyree, E. B. and John, E. S.—(1947) *Amer. J. Physiol.*, **150**, 480.
Soanes, W. A. and Dodson, C. C.—(1954) *J. Urol.*, **72**, 705.
Ungar, F., Rosenfeld, G., Dorfman, R. I. and Pincus, G.—(1955) *Endocrinology*, **56**, 30.—(1955) *Ibid.*, **56**, 24.
van Cauwenberg, H., Fischer, P., Viliens, M. and Bacq, Z. M.—(1957) *C. R. Soc. Biol., Paris*, **151**, 198.
Venters, K. D. and Painter, E. E.—(1951) *Fed. Proc.*, **10**, 141.
Wexler, B. C., Pencharz, R. and Thomas, S. F.—(1952) *Proc. Soc. exp. Biol. N.Y.*, **79**, 183.